# ASO Author Reflections: Importance of Preoperative Prediction of Survival in Resectable Pancreatic Ductal Adenocarcinoma

**DOI:** 10.1245/s10434-024-15853-1

**Published:** 2024-07-17

**Authors:** Takanori Konishi, Shigetsugu Takano, Masayuki Ohtsuka

**Affiliations:** Department of General Surgery, Chiba University Graduate School of Medicine, Chiba, Japan

## Past

Multidisciplinary treatment is crucial to prolong the survival of patients with pancreatic ductal adenocarcinoma (PDAC). Adjuvant chemotherapy has been highly established; however, the efficacy of neoadjuvant therapy for resectable PDAC remains controversial.^1,2^ The Prep-02/JSAP-05 trial revealed the survival benefits of neoadjuvant chemotherapy with gemcitabine and S-1 for resectable PDAC.^3^ Conversely, effect of neoadjuvant therapy could differ according to the disease progression,^4^ and optimal candidates of neoadjuvant therapy for resectable PDAC have not been well determined. Survival prediction upon diagnosis may help determine the treatment strategy for resectable PDAC. To date, previous studies have revealed several clinical factors that are associated with patient’s survival. However, no single preoperative marker is sufficient to accurately predict postoperative survival for resectable PDAC.

## Present

In the current study,^5^ we retrospectively analyzed the oncological outcome of patients with resectable PDAC who underwent upfront surgery with planned adjuvant chemotherapy. In this cohort, 24.9% of the patients did not have tumor recurrence within 5 years after upfront surgery and 5-year overall survival rate was 33.3%. Multivariate analyses revealed that tumor diameter in computed tomography ≤ 19 mm, s-pancreas-1 antigen (span-1) ≤ 30 U/ml, prognostic nutritional index (PNI) ≥ 44.31, and lymphocyte-to-monocyte ratio (LMR) ≥ 3.79 were independent prognostic factors for preferable overall survival. A prognostic prediction model was developed on the basis of the four preoperative prognostic factors for the accurate prediction of long-term survival before treatment. This model extracted the patient’s cohort who experienced good oncological outcomes with a 5-year overall survival rate of 82.4% even without neoadjuvant therapy. This study indicated that anatomical, biological, nutritional, and inflammatory factors evaluation improve the quality of prognostic prediction before treatment and may be valuable for introducing an ideal multidisciplinary treatment for resectable PDAC.

## Future

The clinical benefits of neoadjuvant therapy for resectable PDAC need to be further clarified. In addition, future research should focus on optimal candidates for neoadjuvant therapy as well as the ideal regimen or duration of neoadjuvant therapy for resectable PDAC. The effect of neoadjuvant therapy on survival is theoretically minimal for patients having long-term survival after upfront surgery with adjuvant therapy; thus, the accurate survival prediction before treatment will facilitate the decision-making process for providing neoadjuvant therapy for patients with resectable PDAC. Validating our results in a prospective study with larger cohort of patients is warranted in the future for the clinical use of the prognostic prediction model in this study. In addition, the effect of neoadjuvant therapy on the oncological outcomes after pancreatectomy according to the prognostic prediction model should be clarified.
